# Immunomodulatory Effects Mediated by Serotonin

**DOI:** 10.1155/2015/354957

**Published:** 2015-04-19

**Authors:** Rodrigo Arreola, Enrique Becerril-Villanueva, Carlos Cruz-Fuentes, Marco Antonio Velasco-Velázquez, María Eugenia Garcés-Alvarez, Gabriela Hurtado-Alvarado, Saray Quintero-Fabian, Lenin Pavón

**Affiliations:** ^1^Psychiatric Genetics Department, Clinical Research Branch, National Institute of Psychiatry, “Ramón de la Fuente”, Calzada México-Xochimilco 101, Colonia San Lorenzo Huipulco, Tlalpan, 14370 Mexico City, DF, Mexico; ^2^Department of Psychoimmunology, National Institute of Psychiatry, “Ramón de la Fuente”, Calzada México-Xochimilco 101, Colonia San Lorenzo Huipulco, Tlalpan, 14370 Mexico City, DF, Mexico; ^3^School of Medicine, National Autonomous University of Mexico, Avenida Universidad 3000, Coyoacan, 04510 Mexico City, DF, Mexico; ^4^Area of Neurosciences, Department of Biology of Reproduction, CBS, Universidad Autonoma Metropolitana, Unidad Iztapalapa, Avenida San Rafael Atlixco No. 186, Colonia Vicentina, Iztapalapa, 09340 Mexico City, DF, Mexico; ^5^Genetics Unit Nutrition of Biomedical Research Institute of Universidad Nacional Autónoma de México at Instituto Nacional de Pediatría, Avenida del Iman No. 1, cuarto piso, Colonia Insurgentes-Cuicuilco, Coyoacan, 04530 Mexico City, DF, Mexico

## Abstract

Serotonin (5-HT) induces concentration-dependent metabolic effects in diverse cell types, including neurons, entherochromaffin cells, adipocytes, pancreatic beta-cells, fibroblasts, smooth muscle cells, epithelial cells, and leukocytes. Three classes of genes regulating 5-HT function are constitutively expressed or induced in these cells: (a) membrane proteins that regulate the response to 5-HT, such as SERT, 5HTR-GPCR, and the 5HT_3_-ion channels; (b) downstream signaling transduction proteins; and (c) enzymes controlling 5-HT metabolism, such as IDO and MAO, which can generate biologically active catabolites, including melatonin, kynurenines, and kynurenamines. This review covers the clinical and experimental mechanisms involved in 5-HT-induced immunomodulation. These mechanisms are cell-specific and depend on the expression of serotonergic components in immune cells. Consequently, 5-HT can modulate several immunological events, such as chemotaxis, leukocyte activation, proliferation, cytokine secretion, anergy, and apoptosis. The effects of 5-HT on immune cells may be relevant in the clinical outcome of pathologies with an inflammatory component. Major depression, fibromyalgia, Alzheimer disease, psoriasis, arthritis, allergies, and asthma are all associated with changes in the serotonergic system associated with leukocytes. Thus, pharmacological regulation of the serotonergic system may modulate immune function and provide therapeutic alternatives for these diseases.

## 1. Introduction

Serotonin (5-HT), also known as 5-hydroxytryptamine or 3-(2-aminoetil)-1H-indol-5-ol, is a monoamine containing two nitrogen molecules: the first nitrogen is basic and embedded within the indol-5-ol; the second, within 2-aminoethyl, is located at the terminus of the aliphatic chain. 5-HT is generated from tryptophan and serves as a substrate for the synthesis of a diverse set of molecules, such as melatonin, formyl-5-hydroxykynurenamine, and 5-hydroxyindoleacetic acid [1]. In addition, 5-HT is a signaling molecule that affects the immune [2], gastrointestinal [3], and nervous [4] systems in paracrine, endocrine, and juxtacrine fashion. Finally, 5-HT regulates development during cellular differentiation and ontogeny (morphogenesis) in several cell linages [5–7].

The majority of 5-HT synthesis, up to 90%, takes place in gastrointestinal enterochromaffin (EC) cells, followed by synthesis in myenteric neurons (5%) and the brain [8, 9]. In the 1980s, 5-HT was identified as an immunomodulator for its ability to stimulate or inhibit inflammation [10]. This immune regulation—which has yet to be fully elucidated—is orchestrated by the serotonergic system. Therefore, to understand disease pathologies related to the immune system, it is important to consider the function of serotonergic components. Specifically, insight can be gained by understanding how serotonergic components are related to mechanisms of immune modulation that depend on 5-HT receptors (5HTR) expression in leukocytes and other cells involved in an inflammatory response.

## 2. A Brief History of 5-HT Discovery

The discovery of 5-HT was a product of collaborative endeavors initiated in the last quarter of the 19th century [11] that lasted into the second half of the 20th century. Initial studies identified an extract with vasoconstriction properties from a platelet fraction of uncoagulated blood [12]. In research conducted in Rome during the 1930s, Vittorio Erspamer isolated a molecule from gastrointestinal EC cells with the capacity to generate smooth muscle contractions in a rat uterus. Chemical analysis identified the molecule as an indoleamine and it was named enteramine [13]. During the 1940s in the Cleveland Clinic research department, Maurice Rapport, Arda Green, and Irving Page purified and characterized a vasoconstrictor compound generated shortly after coagulation and related to hypertension. In a tour de force, the molecule was purified from 900 liters of serum obtained from 2 tons of bull's blood [14, 15]. The name serotonin emerged after the substance was crystallized in 1948 because it was obtained from serum (“ser”) and could induce vascular tone (“tonin”) in blood vessels [16]. Subsequently, the crystalline vasoconstrictor substance was shown to be a single complex composed of creatinine and indol-derivates, which permitted a structural model of 5-HT based on UV-spectrophotometry [17]. Chemical synthesis of 5-HT by Hamlin and Fischer in 1951 [18] provided significant progress allowing for the confirmation of its pharmacological effects [19] and a comparison with the previously isolated enteramine [20]. Interest in understanding the physiological role of 5-HT prompted efforts to isolate the compound from different mammals and tissues, such as the central nervous system [21].

Since the 1970s there has been an established association between the serotonergic system and affective disorders as well as mood changes [22]. Recently, serotonin has been associated with a myriad of processes [23], including aggression [24], sleep [25], appetite [26], pain [27], bone density [28], tissue regeneration [29], platelet aggregation [30], and gastrointestinal function [31]. The influence of 5-HT on the immune system has also been recognized, although the specific mechanisms underlying these effects are not completely understood and may require confirmation in human cells. Despite these pitfalls, it is well acknowledged that the serotonergic system and associated molecules expressed in immune cells can influence mood disorders, such as major depression [32] and schizophrenia [33, 34].

## 3. Components of the Serotonergic System Are Expressed in Leukocytes

The expression of serotonergic components is differentially regulated between tissues and cell types. While the expression and function of serotonergic proteins has primarily been studied within the central nervous system [81], it should be pointed out that no functional differences between cell types have been identified. The serotonergic components expressed in the immune system encompass a complex ensemble of proteins that coordinate the synthesis and degradation [1], transport and storage [58], and response to 5-HT stimulation [40]. In leukocytes, the expression of serotonergic components (Table 1) is modulated by the concentration of extracellular and intracellular 5-HT. Furthermore, the signals generated by 5-HT interactions with leukocytes are distinct depending on function, developmental stage, and activation status of the cell. This functional heterogeneity suggests that the serotonergic system can precisely regulate a wide range of immunomodulatory effects [81].

### 3.1. Catabolism and Anabolism of 5-HT

**Figure 1:**
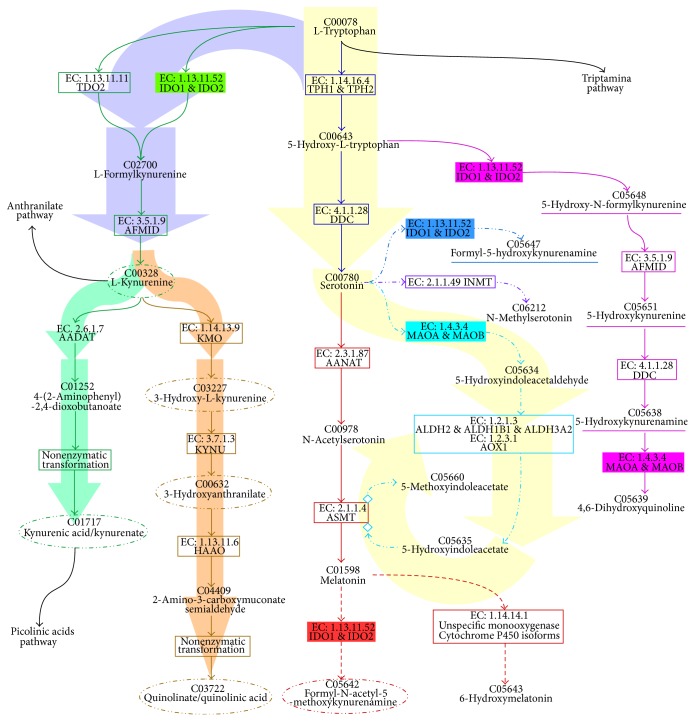
Metabolic pathways associated with 5-HT. The metabolic pathways branching from the catabolism of tryptophan are shown. Green and brown branches show kynurenine pathways from tryptophan. The dark blue branch displays the 5-HT generation pathway while the red branch displays the melatonin generation pathway (solid line) and the melatonin catabolism pathway (dashed lines). The 5-HT catabolism pathways are depicted in blue, purple, and cyan dotted lines. The most relevant compounds are circled with dotted lines and underlined compounds have no demonstrated effects. An additional catabolic pathway marked in pink from 5-hydroxy-L-tryptophan generates 5-hydroxy-kynurenine and 5-hidroxy-kynurenamine family compounds (paths C05648 and C05647), which have no demonstrated biological effects. The compounds in these pathways are denoted by their “Kyoto Encyclopedia of Genes and Genomes” (KEGG) code (http://www.genome.jp/kegg/). Enzymes with their classification codes (EC, http://www.chem.qmul.ac.uk/iubmb/enzyme/) and UNIPROT gene names are shown in squares. The shading arrows show the most studied pathways.

The essential amino acid Tryptophan is utilized by many cell types and can be converted into a wide range of chemically related products, among the best known are 5-HT and melatonin, but also include kynurenines and kynurenamines (Figure 1). In macrophages and T lymphocytes the indoleamine 2,3-dioxygenase (IDO1 & IDO2. EC: 1.13.11.52) [60, 82] enzymes help degrade tryptophan to generate kynurenines and produce kynurenamines from 5-HT or melatonin [83]. In general, all of these compounds can modulate immune responses [1, 84–86]. However, the mechanisms by which these molecules exert an immunomodulating function are not completely elucidated. Some observations suggest that kynurenines and kynurenamines function in negative feedback loops to modulate 5-HT-mediated inflammation, other proinflammatory molecules, and melatonin levels.

#### 3.1.1. Anabolism

The synthesis of serotonin begins with the essential amino acid, tryptophan, and follows two-enzymatic steps. First, a hydroxyl group is added by* tryptophan* 5-*hydroxylase* (TPH; EC: 1.14.16.4) to generate 5-hydroxytryptophan. Mammals produce two TPH enzymes encoded by two independent genes, TPH1 and TPH2. While TPH1 is expressed in peripheral tissues, TPH2 is exclusively expressed in the central nervous system [87–89]. After this hydroxylation step, a carboxyl group is removed by an aromatic L-amino acid decarboxylase (DDC; EC: 4.1.1.28) generating 5-HT [90–92].

#### 3.1.2. Catabolism

Within the immune system, four catabolic pathways for the breakdown of 5-HT have been observed. One pathway begins with the generation of melatonin from 5-HT through two enzymatic steps; first, 5-HT is acetylated by arylalkylamine N-Acetyltransferase (AANAT; EC: 2.3.1.87) generating N-acetyl 5-HT, which then acquires a methyl group from N-acetylserotonin-O-methyltransferase (ASMT; EC: 2.1.1.4; previously known as hydroxyndole-O-methyltransferase, HIOMT) to become melatonin [93, 94]. Subsequently, the enzyme indoleamine 2,3-dioxygenase (IDO1 & IDO2; EC: 1.13.11.52) can convert melatonin into a cyclooxygenase (COX; EC: 1.14.99.1) inhibitor called formyl-N-acetyl-5-methoxykynurenamine. Interestingly this metabolite can function to block the synthesis of prostaglandins [95], yet other melatonin metabolites, including 5-methoxyindole acetic acid and 6-hydroxymelatonin, have no reported function [96].

A second catabolic pathway of 5-HT utilizes the enzyme indoleamine 2,3-dioxygenase (IDO) and generates formyl-5-hydroxykynurenamine. In a third pathway, 5-HT is transformed into 5-hydroxyindoleacetic acid with monoamine oxidase A/B (MAO-A o MAO-B; EC: 1.4.3.4) among other enzymes. Interestingly, MAO-A expression, which is regulated by the cytokines IL-4 and IL-3, has been identified in human monocytes from peripheral blood [62]. A fourth catabolic pathway generates N-methylserotonin from 5-HT using the enzyme amine N-methyltransferase (INMT; EC 2.1.1.49) and may also be active in immune cells.

It is not thoroughly understood that the extent by which the byproducts and metabolites generated during catabolism may affect the immune system [1, 84]. The identification of additional 5-HT metabolites in plasma, including serotonin-O-sulfate [97] and 5-hydroxykynurenamine [98], suggests that other catabolic pathways linked to specific biochemical processes, such as activation and cell proliferation, may also be associated with the modulation of the immune system.

One additional catabolic pathway related with the four previously described utilizes the enzyme IDO to generate kynurenines from tryptophan. This pathway is positively regulated when immune cells become activated and begin secreting IFN-*α*, IFN-*β* e IFN-*γ*, TNF-*α*, TGF-*β*, IL-1*β*, and IL-2 [85, 99–101], which significantly consumes tryptophan and limits its availability for 5-HT production. L-kynurenine, 3-hydroxy-L-Kynurenine, and 3-hydroxyanthranilic acid can negatively modulate immune responses. Specifically, Vécsei and coworkers noted the blockage of cell proliferation as well as the potential induction of apoptosis in Th1 and NK cells [85, 101–103].

It was recently proposed that 5-hydroxyindole thiazolidine carboxylic acid, a 5-HT byproduct found in the intestinal tissues and several brain regions of rats, is generated from the condensation of 5-hydroxyindole acetaldehyde and L-cysteine by a carbon-sulfur lyase (EC 4.4.1.). However, evidence supporting this enzymatic condensation remains to be confirmed. In addition the properties of this byproduct or its potential influence over immunological cells remain to be investigated [104].

### 3.2. 5-HT Receptors: (5HTR)

5-HT modulates many leukocyte functions ranging from activation of the immune response to memory cell generation. The effects mediated by 5-HT are dependent on the differential expression of serotonergic components in leukocytes. For example, serotonin receptors (5HTR) on immune cells influence cytokine proliferation, delivery, migration, and cellular activation. Signaling through the 5HTR affects chemoattraction in immature mammalian dendritic cells (human and rodent) but not in mature cells, which respond to 5-HT by secreting IL-6 [43]. In addition, 5HTR signaling influences naïve T cell activation in mice by activating 5HT_7_ [40] and regulates lymphocyte B cell proliferation through 5HT_1A_ [41].

#### 3.2.1. 5HTR Are G Protein-Coupled Receptors (GPCR)

The 5HTR belong to the GPCR family class A, also known as 7-transmembrane domain (7TM) receptors. GPCR are classified into 6 classes according to a database from the International Union of Basic and Clinical Pharmacology (IUPHAR: http://www.guidetopharmacology.org/) [105]. This system includes classes A, B, and C, as well as the adhesion,* Frizzled*, and other 7TM classes. Receptors for adenosine, adrenaline/noradrenaline, 5HT_1_, 5HT_2_, 5HT_4_, 5HT_5_, 5HT_6_, and 5HT_7_ all belong to GPCR class A. Furthermore, the 5HTRs are comprised of 6 families and 13 subfamilies [106] with an undetermined number of isoforms that may be produced by alternative splicing. This receptor diversity suggests that a great amount of functional variation may exist between the 5HTRs [107].

Signal transduction from 5HTR is similar to standard GPCRs. G proteins form heterotrimeric complexes made of the subunits G*α*, G*β* and G*γ*; the complex is coupled to the C-terminus of the transmembrane 5HTR. Different subtypes of the G*α* proteins (G*α*_i/o_, G*α*_s_, or G*α*_q11_) may generate different transduction responses functioning either as activators or inhibitors. When the 5-HT ligand binds its receptor to induce signal transduction it elicits a conformational change in the receptor to facilitate activity [81]. Signal transduction can be carried out by the effector proteins G*α* or the heterodimer G*β*-G*γ*. While transduction mechanisms have been better depicted for G*α*, especially in cells of the nervous system [108], recent work has been devoted to the role of G*α* and G*β*-G*γ* in the immune system [109, 110].

In the last few years, a number of investigations have demonstrated that GPCR are capable of assembling dimers (homo and heterodimers) as well as oligomers. Receptors 5HT_1B_ and 5HT_1D_ can assembly into homodimers and heterodimers when coexpressed in the same cell. Notably, the receptors display roughly 77% sequence identity within the 7TM domain [111]. The 5HT_2C_ receptors form homodimers within the cellular membrane [112–114] and it has been proposed that signaling is initiated when two 5-HT molecules bind the dimer [115]. However, activation a single subunit within the 5HT_4_ homodimer is sufficient to initiate G protein activation even though simultaneous activation of both receptors doubles the activation efficiency of the pathway. While it appears that 5HTR tend to form homodimers rather than heterodimers, the latter possibility has not been discarded [116].

Milligan and coworkers have postulated that the formation of heterodimers could generate specificity between 5HTR and its substrates, which could be especially relevant for the use and design of pharmacological agents [117]. Despite current knowledge suggesting that the 5-HT receptors function as homodimers and maybe heterodimers, it is possible that higher subunit organizations (e.g., trimers and tetramers) could function under certain circumstances [114, 118].

To better understand 5-HT cellular-mediated processes, it will be necessary to characterize the quaternary structure and stoichiometry of 5HTR during oligomerization in regular cellular processes. It will also be important to determine the biochemical details of the quaternary transitional structure (quinary structure) of 5HTR to establish not only its functional characteristics, but also the coupling and assembly with cytoskeleton proteins. Recently, the crystal structures of the receptor-agonist complexes, 5HT_1B_ and 5HT_2B_ with ergotamine and dihydroergotamina, respectively, were reported and provided structural information to better understand receptor-ligand interactions and agonist selectivity, which could inform 5HTR-based drug design [119, 120].

#### 3.2.2. The 5HT_3_ Receptors Form a Cationic Channel

The 5HT_3_ receptors are part of a cation-selective ion channel Cys-loop superfamily and have been detected in T lymphocytes, monocytes [47], mature dendritic cells [43], and mast cells [38]. The functional unit of 5HT_3_, a pentameric ring, generates a central ion channel and can be composed of two 5HT_3A_ and three 5HT_3B_ subunits [121, 122]. Each subunit contains a large N-terminal domain with the 5-HT binding site and four transmembrane domains connected with intracellular and extracellular loops [123–125]. The 5HT_3_ receptors are regulated through protein modifications and cytoskeletal rearrangements, dependent on protein kinases (A and C) and F-actin, respectively [123, 126].

There are a number of 5HT_3_ receptor variants that can be expressed from the human genome. The genes encoding 5HT_3A_ and 5HT_3B_ are located on chromosome 11q23, and those encoding 5HT_3C_, 5HT_3D_, and 5HT_3E_ are on chromosome 3q27. However, the total number of isoforms generated from these genes by alternative splicing has yet to be determined [127–130]. Interestingly, the subunits 5HT_3C_, 5HT_3D_, 5HT_3E_, and 5HT_3Ea_ are not sufficient to form functional pentamers but can generate them with 5HT_3A_ and hence modify 5-HT responses [131].

In the context of the central nervous system, 5HT_**3**_ receptors are associated with rapid activation and inhibition responses in addition to fast cellular depolarization [49, 132]. The cellular depolarization response is unique to neurons, as this has not been observed in immune cells. In neurons, 5HT_3_ receptors modulate the delivery of neurotransmitters, such as dopamine [133], whereas the same receptors elicit the release of cytokines from immune cells. Human lymph nodes preferentially express the 5HT_3A_ and 5HT_3E_ variants [49]. In addition, 5HT_3A_ is expressed in naïve and activated B-lymphocytes [48], T lymphocytes, and human monocytes, but expression has not been detected from monocyte-derived dendritic cells [47]. Inhibiting the 5HT_3_ receptors with antagonists, such as ondasetron and tropisentrol, disrupts TNF-*α* and IL-1*β* production, suggesting that these receptors may activate the p38/MAPK pathway [134, 135].

### 3.3. SERT: 5-HT Transporter

The serotonin transporter SERT actively moves extracellular 5-HT across the plasma membrane into the cell. The transporter is also known as* solute carrier family 6, member 4* (SLC6A4), and belongs to a family of neurotransmitters with 12 transmembrane domains. The function of SERT in platelets is critical for maintaining adequate 5-HT concentrations in the circulatory system [136]. To function, SERT depends on the transport of Na^+^/Cl^−^ ions, yet the coordinated mechanism of 5-HT and ion transport remains to be elucidated.

The gene encoding SERT has 14 exons and is located on chromosome 17q11.1-17q12. Two genetic polymorphisms in the gene regulatory region modulate transcription generating a complex mix of long and short variants [137]. The SERT functional unit is a dimer but it has been suggested that two SERT homodimers could assemble into a tetramer [138]. Other members of the protein family form heterodimers; however, these may not be functional [139]. Therefore, SERT homodimers are currently accepted as the biochemically functional unit.

SERT dimer formation relies on several posttranslational modifications. The SERT proteins are glycosylated, and then sialic acid is inserted into each of two N-linked glycans. In the absence of glycosylation, the functional activity of SERT is reduced. Furthermore, the addition of sialic acid molecules is important for dimer formation and the association with myosin IIA (a kinase that anchors protein kinase G, PKG) at the cytoskeleton. This association can regulate SERT phosphorylation by the guanosine monophosphate-dependent PKG [140].

A complex regulatory mechanism of SERT internalization associated with the cytoskeleton has been described in platelets and is referred to as serotonylation (see Box 1). The process of serotonylation depends on the 5-HT gradient between the extracelluar and intracellular space created by SERT activity. The gradient mediates regulation via cytoskeletal components, vimentin, 5HTR, SERT, small GTPases, transglutaminases (TGases), and possibly p21 activated kinases (p21/PAK) [141]. Specifically, increased levels of 5-HT activate SERT internalization through activation of small GTPases by serotonylation (TGases covalently linking the 5-HT to the small GTPases). Currently efforts are being directed to the characterization of SERT internalization mechanisms in leukocytes.

**Box 1 (serotonylation: SERT, TGasas, and small GTPases).**

Serotonylation is the process by which 5-HT is covalently bound to a protein through a transamination reaction and constitutes a mechanism for regulating signal transduction. This process requires high intracellular levels of 5-HT and is mediated by transglutaminases (TGases. EC: 2.3.2.13). Physiological serotonylation has been demonstrated in platelets and other cells [142, 143] but not in leukocytes. However, serotonylation may be involved in specific leukocyte functions required for chemotaxis or cytokine secretion [43, 45] because this modification regulates similar functions in platelets and pancreatic beta cells.

Although many cytoplasmic proteins can be serotonylated, the effect of serotonylation on small GTPases during platelet activation and aggregation is noteworthy [142]. Serotonylation induces constitutive RhoA activation and, consequently, the cytoskeletal reorganization needed for aggregation [144–147]. Increased intracellular Ca^2+^ and 5-HT in platelets also activates Rab4-mediated exocytosis of alpha granules by Ca^2+^-dependent TGase-mediated serotonylation [142].

In pancreatic beta cells, Rab3a and Rab27a are serotonylated during insulin exocytosis [143]. In smooth muscle cells TGase-2-mediated serotonylation of RhoA may be required for proliferation [148–150]. Furthermore, serotonylation of RhoA not only constitutively activates it, but also increases its rate of proteosomal degradation [148]. Since Rab27a and Rab27b also participate in exocytosis from platelets and endocrine cells [151–155], these processes may be also susceptible to serotonylation.

Additionally, 5-HT induces vimentin filament reorientation around SERT [156]. Furthermore, serotonylation of Rab4 promotes interaction with the SERT C-terminal domain regulating translocation [157]. Importantly, there are no reports that specifically study serotonylation in leucocytes, but available information suggests that it may regulate cytoskeletal reorganization in these cells [43, 45]. Rab27a is expressed in cytotoxic T cells and regulates the last step of granular exocytosis [158, 159]. Rab27, RhoA, and Rab4 are also expressed in several cells of the immune system [160–162]; therefore it is possible that serotonylation regulates exocytosis or cytoskeletal reorganization during functions such as MHC presentation.

### 3.4. 5-HT Storage and Exocytosis in Leukocytes

Three potential outcomes may take place following 5-HT internalization: signaling may be activated by serotonylation, 5-HT may undergo enzymatic transformation, or 5-HT may be stored in specialized vesicles. The synthesis, storage, and transport of 5-HT in the immune system are more diverse and complex than previously reported, and a seminal review of the subject was recently published by Ahern in 2011 [163].

Storage of 5-HT in the immune system allows for its reuse by exocytosis, which occurs in dendritic cells, peripheral blood lymphocytes, and platelets. Within these cells, the vesicular monoamine transporter (VMAT) is responsible for storing 5-HT inside dense granules [164]. The 5-HT transport and storage/exocytosis pathways in platelets are coupled to the serotonylation signaling pathway, which assesses 5-HT concentrations and dictates its fate [165]. LAMP1 containing vesicles in monocyte-derived dendritic cells from mice also store 5-HT, and upon ATP stimulation (Ca^2+^ influx), these cells secrete 5-HT and cytokines [58]. A major focus of current endeavors is the investigation of several 5-HT mediated processes in other types of leukocytes, including 5-HT vesicle storage, small GTPases-mediated serotonylation, cytoskeletal associations, and the metabolism of 5-HT into derivatives, such as melatonin and kynurenins.

## 4. The Effects of 5-HT on Leukocytes

Innate and adaptive immune responses rely on a diverse set of cell types from lymphoid linage (T cells, B cells, and NK cells), myeloid linage (neutrophils, eosinophils, basophils, monocytes, and mast cells), or myeloid/lymphoid linage (dendritic cells) origins. Professional antigen presenting cells (APC), such as dendritic cells and macrophages, link the innate and adaptive immune responses by recognizing, processing, and presenting antigens on MHC-II. Antigen presentation activates naïve T cells initiating clonal proliferation and generating the immune memory, which is essential for the adaptive phase of an immune response.

The local concentration of 5-HT can modulate a number of events during these immune responses [10]. One reason immune cells respond to 5-HT is that, as mentioned previously, they constitutively express the molecular machinery that constitutes the serotonergic system.

### 4.1. Neutrophils

Neutrophils, the most abundant innate immune cells in human blood, constitute a first line of defense against infection by recognizing foreign antigens, producing antimicrobial compounds, and secreting cytokines and chemokines to recruit immunocompetent cells [166]. Mouse models have demonstrated that neutrophil recruitment to sites of acute inflammation requires platelet-derived chemotactic stimuli, such as 5-HT, PAF (platelet-activation factor), and histamine [167, 168].

### 4.2. Monocytes/Macrophages

Macrophages as well as their precursors, circulating monocytes, participate in immune responses during pathogen infection. Monocytes can be divided in two subsets: those that express CD14, a component of the lipopolysaccharide (LPS) receptor complex, and those that express CD16, the Fc*γ*RIII immunoglobulin receptor [169, 170]. CD14+ cells, which constitute 80–90% of the circulating monocytes, express 5HT_1E_, 5HT_2A_, 5HT_3_, 5HT_4_, and 5HT_7_ mRNAs [42]. LPS stimulation does not affect 5HTR expression suggesting that the receptors constitutively regulate cell functions.

The addition of 5-HT to monocytes induces phagocytosis of opsonized goat erythrocytes [171]. However, 5-HT has been noted to elicit a number of responses in CD14+ cells isolated from peripheral blood of healthy subjects [42]: (i) 5-HT signaling decreases TNF-*α* secretion in a dose-dependent manner; (ii) 5-HT signaling enhances LPS-induced secretion IL-12p40 from activated monocytes, which acts as a chemoattractant for macrophages and promotes the migration of bacterially stimulated dendritic cells; and (iii) 5-HT signaling enhances LPS-induced secretion of IL-6, IL-1*β*, IL-8/CXCL8. The first two effects are mediated by the 5HT_4_ and 5HT_7_ receptors, whereas the third effect requires 5HT_3_, 5HT_4_, or 5HT_7_.

Macrophages also respond to 5-HT although reports conflict as to whether the response is inhibitory or stimulatory and the mechanisms involved have yet to be clearly described. For example, combined stimulation with 5-HT and muramyl peptides induces superoxide secretion by peritoneal macrophages [172]. Bovine alveolar macrophages release chemotactic factors for neutrophils and monocytes in response to 5-HT and histamine [173]. Similarly, murine macrophages detect 5-HT with the 5HT_2C_ receptor to induce the secretion of CCL2, which induces monocyte migration [174]. In peritoneal murine macrophages 5-HT induces phagocytosis in a dose-dependent manner through 5HT_1A_ and NF*κ*-B activation [175].

On the other hand, 5-HT is also reported to function as negative regulator. For example, 5-HT_2_ signaling limited the activation of murine macrophages stimulated* in vitro* with high concentrations of IFN-*γ* [176]. Furthermore, human alveolar macrophages stimulated with LPS and 5-HT secreted less TNF-*α* and IL-12, but more IL-10, nitric oxide, and prostangladin-E2. However, the receptors mediating these effects have yet to be described [177].

The different macrophage responses elicited by 5-HT may be due to phenotypic differences in tissue-specific macrophages (i.e., changes in the proportion of 5-HT receptors) or the effect of cooperative signaling with other molecules [178]. Finally, macrophages can rapidly metabolize 5-HT to 5-hydroxyindole acetic acid [179], a biotransformation pathway that may be mediated by MAO-A/B, ALDH/AOX and ASMT, which could affect serotonergic responses in these cells.

### 4.3. Dendritic Cells (DCs)

DCs play a crucial role in the immune response to infectious pathogens. In humans, circulating DCs characteristically expresses high levels of class II HLA molecules and are proficient in antigen uptake and processing. However, they express low levels of HLA class I and costimulatory molecules, such as CD80 and CD86, and lack common lineage markers such as CD3, CD14, CD16, CD19, CD20, and CD56. DCs are positioned between the adaptive and innate immune systems: detecting microbial infection, tissue damage, and inflammatory signals to promote the activation of antigen-specific responses [180–182].

Peripheral blood monocytes can differentiate into macrophages or DCs depending on environmental stimuli. Culturing cells with IL-4 and granulocyte-macrophage colony-stimulating factor (GM-CSF) induces human monocytes and murine myeloid progenitors to differentiate into monocyte-derived dendritic cells (MDDCs) and bone marrow dendritic cells (BMDCs), respectively. These cells, frequently used as models for dendritic cell biology [50, 58, 183], are sensitive to LPS and 5-HT (Table 2).

When 5-HT is added during IL-4/GM-CSF differentiation, the resulting MDDCs display lower levels of CD1a, CD86, and HLA-DR but had increased CD14 expression. However, other markers such as CD40, CD80, or CD83 were unaffected. Murine BMDCs can be matured by LPS stimulation to generate cells with characteristics of DCs [184]. Although immature (CD11c+CD86−) and mature (CD11c+CD86+) BMDCs constitutively express SERT, maturation induced by LPS increases SERT expression and, consequently, mature BMDCs have an increased capability for intake, storage, and exocytosis of 5-HT [58]. BMDC maturation also reduces the expression of enzymes involved in the metabolism of 5-HT, such as MAO-A and -B. Furthermore, immature and mature dendritic-like cells respond differently to 5-HT [50]. In mature MDDCs, the 5HT_3_ receptor contributes to changes in intracellular Ca^2+^ concentration required for the secretion of IL-8 and IL-1*β* [2]. On the other hand, 5-HT inhibits CXCL10 secretion from mature MDDC, but CCL22 secretion is not affected [43].

Additionally, 5-HT can regulate MDDC functional responses. Costimulation with 5-HT and LPS induces immature MDDCs migration in a 5HT_1B_ and 5HT_2A_-dependent manner. However, if 5-HT is added subsequent to LPS-mediated maturation, migration is unaffected but cytokine and chemokine secretion is induced [43]. Additional* in vitro* experiments with MDDCs and BMDCs have also demonstrated that 5-HT activates the secretion of pro-inflammatory cytokines [2, 185].

One key DC function is the activation of T cells and 5-HT can also regulate this fundamental immunological process. Since dendritic-like cells do not express TPH1, it is unlikely that they can synthetize 5-HT. Therefore, O'Connell and coworkers postulated that SERT-expressing DCs are able to internalize 5-HT from the microenvironment [58]. When interacting with T cells, MDDCs transiently release Ca^2+^, which promotes cytokine and 5-HT secretion from LAMP1+ vesicles [186]. Thus, DCs may internalize and store 5-HT to release into the immunological synapse during T cell activation [58]. Activated T cells express TPH1 [58] and therefore, can synthetize 5-HT. The synthesis of 5-HT in activated T cells is related to tryptophan metabolism [40] and may also be important when T cells interact with target cells [43].

Taken together, 5-HT affects DC differentiation and maturation as well as the profile and function of soluble mediators these cells express. Thus, 5-HT may participate in the generation of a specific subset of DCs with unique immunomodulatory properties [50].

### 4.4. T Cells

Differentiation, proliferation, and the functional responses of T cells can each be modulated by the serotonergic system. Based on experiments using the cell line K562, 5-HT participates in T cell maturation in lymphoid organs in a Na^+^-coupled, 5-HT active transport-dependent manner (presumably SERT), which requires intracellular Ca^2+^ changes [187].

Based on the effects of 5-HT receptor-specific agonists or antagonists, T cell proliferation and secretion of proinflammatory cytokines, such as IL-2 and IFN-*γ*, requires activation of 5HT_1A_ but not 5HT_1C_ [37]. Naïve T cells primarily express 5HT_7_ and low levels of TPH1, but they do not express 5HT_1B_ or SERT. After 5-HT stimulation, the ERK-1,-2/NF-*κ*B pathway is activated in proliferating T cells, and they express 5HT_1B_, 5HT_7_, 5HT_2A_, and TPH1 [40]. In activated human CD4+ T cells, 5-HT or 5HT_3_ specific agonists impair migration towards CXCL12 gradients, but not to those of CCL2 or CCL5, which control T cell migration into tissues. However, immature murine CD4+ T cells do not express 5HT_3_, and 5-HT does not affect cell migration. But, activation of these cells induces the expression of 5HT_3A_ [46] suggesting they may respond to 5-HT once activated.

Therefore, the T cell activation state and environment may influence the effects of 5-HT. For example, 5-HT inhibits phytohemagglutinin- (PHA-) mediated lymphocyte proliferation, possibly through reduced expression and distribution of the IL-2 receptor [188, 189]. In addition, concanavalin A (ConA) and low concentrations of 5-HT increased murine T cell proliferation although the activation of CD4+ and CD8+ subsets was reduced [190, 191]. This demonstrates that 5-HT elicited effects are concentration dependent and suggests: (i) 5-HT induces dose-dependent phenotypes, (ii) differentiation may be achieved by receptors with different 5-HT affinity, and (iii) local 5-HT concentrations are tightly regulated to induce specific effects.

T cells express SERT and, therefore, can acquire 5-HT [51, 52, 192, 193]. However, naïve T cells have reduced SERT functional activity [164] and may result to 5-HT synthesis. In agreement with this hypothesis, a report from Aune and coworkers demonstrates that the inhibition of the 5-HT synthesis in IL-2-stimulated T cells blocks cell proliferation. The addition of 5-hydroxtriptophan, a 5-HT precursor, restores proliferation, further suggesting that these cells synthesize the molecule rather than acquire it [37]. However, further research is required to understand how 5-HT synthesis is regulated in T cells.

### 4.5. B Cells

B cells recognize circulating antigen; as a consequence, they activate processes that end in the generation of memory B cells or antibody-forming plasma cells [194]. 5-HT increases the proliferation of murine B cells by activating 5HT_1A_ [41]. In addition, SERT expression is proportional to the proliferation rates of human leukemic B cells. Specifically, SERT-specific inhibitors, such as fluoxetine, fenfluramine, or 3,4-methylenedioxymethamphetamine (MDMA), elicited anti-proliferative and pro-apoptotic effects [195].

### 4.6. NK Cells

NK cells recognize antigen in the context of CD1 controlling viral replication early in infection and inhibiting the development of cancer [196, 197]. These cells are inhibited with oxidation produced by autologous monocytes and with apoptosis induced by reactive oxygen species (ROS); however, 5-HT signaling limits these forms of inhibition [198]. NK cells express 5-HT_1A_ and therefore can sense 5-HT local concentration [199–202]. In fact, 5HT_1A_-specific antagonists, such as pindobind, exacerbate the inhibitory effect of monocyte-mediated ROS production on NK cells [202].

### 4.7. Eosinophils

Eosinophils are responsible for fighting multicellular parasites and other infections in vertebrates; they also control mechanisms associated with allergy and asthma. Eosinophils express the 5-HT receptors 5HT_1A_, 5HT_1B_, 5HT_1E_, 5HT_2A_, and 5HT_6_, but they do not express 5HT_2C_, 5HT_3_, 5HT_4_, and 5HT_7_. However, differential expression of 5HT_2A_ was detected in allergy and asthma patients [39].

The serum levels of 5-HT are higher in symptomatic asthma patients in comparison to asymptomatic patients, which may influence eosinophil-mediated inflammation in patients with active disease. 5-HT is a potent chemoattractant for eosinophils both* in vivo* and* in vitro* and supports rolling, an important feature of these cells. Antagonists of 5HT_2A_ inhibit both effects suggesting that 5-HT mediates eosinophil activation [45]. Migration and rolling require changes in the actin cytoskeleton and activation of PKC and calmodulin signaling [39, 45], which control the morphological changes required for eosinophil infiltration from circulation to sits of inflammation [39].

### 4.8. Basophils

While basophils represent less than 2% of leukocytes, they actively participate in immune responses in peripheral organs where they are recruited during nematode and ectoparasite infections. They also participate in allergic reactions by releasing histamine in response to specific growth factors, such as IL-3 [203, 204]. In addition, basophils are an important source of IL-4 and therefore may promote Th2 differentiation [205, 206].

The role of 5-HT on basophil functions has not been clarified. Murine basophil exposure to 5-HT inhibits IL-4 secretion in a dose-dependent manner both* in vitro* and* in vivo* [204]. 5-HT also blocks the release of histamine, IL-4, and IL-6 from murine basophils following IL-3 stimulation as well as blocking the release of IL-13 and IL-4 from human peripheral blood basophils [204]. Intraperitoneal administration of IL-33 to mice normally increases the serum levels of IL-4, but is blocked by the administration of 5-HT [204]. Although murine basophils express SERT, drugs targeting the transporter, such as fluoxetine or citalopram (Selective Serotonin Reuptake Inhibitor, SSRI), do not block the effect of 5-HT on cytokine release suggesting that other transporters may be used, such as the organic cation transporter 3 [204].

### 4.9. Mast Cells

Human mast cells play a local regulatory role at the site of inflammation. Human mast cells express 5HT_1A_, 5HT_1B_, 5HT_1E_, 5HT_2A_, 5HT_2B_, 5HT_2C_, 5HT_3_, 5HT_4_, and 5HT_7_. Cell migration and fibronectin adhesion are both influenced by the addition of 5-HT to these cells. Although 5HT_2A_ is the predominant receptor, responses are primarily mediated through 5HT_1A_ and can be blocked by the G-protein inhibitor pertussis toxin [38].

### 4.10. Platelets

Platelets are well known for initiating coagulation and maintaining vascular tone; however, these cells also participate in inflammatory responses by releasing histamine and PAF. They provide a local source of biogenic amines, including 5-HT, in damaged regions of the vasculature [207]. Platelets uptake 5-HT from plasma in a fast and saturable process; therefore, they are also key regulators of the circulatory 5-HT concentration [164]. The local concentration of 5-HT during platelet aggregation is approximately 100 *μ*M [208]. 5-HT uptake is mediated by SERT, and once inside the cell it is transported to dense granules by VMAT (Vesicle Monoamine Transporter) or hydrolyzed by MAO [136].

5-HT signaling activates Rab4, which controls alpha granule secretion, and RhoA, which induces the cytoskeletal reorganization required for adhesion and aggregation [144, 145, 209]. It is reported that Rab4 activation occurs by serotonylation (see Box 1), and it is likely that RhoA is similarly activated.

Increases in the serum levels of 5-HT enhance SERT density on the platelet cell membrane [142, 148, 156, 157]. Some studies suggest that human platelets initiate murine T cell activation by Fc*ϵ*RI-mediated contact sensitivity and the release of 5-HT [207]. However, the functional role that platelet-derived 5-HT plays in the immune system is still far from being fully understood.

## 5. Changes to the Serotonergic System Affect Immune Responses and Have Clinical Implications

Immune cells respond to 5-HT with varying degrees of sensitivity, which can be partially explained by differences in the expression of serotonergic components. In this section, we review how pathologies with reported alterations to the serotonergic system affect the immune system (Table 2). We also discuss the effects of SERT-targeting drugs, such as SSRIs, as well as drugs that target 5-HT receptors (Table 3).

### 5.1. Diseases Associated with Systemically Low 5-HT Levels

Major depressive disorder (MDD), Fibromyalgia, infections, and Alzheimer's disease commonly display reduced 5-HT serum levels. The precise effects these changes have on the immune system in each disease are poorly defined. However, MDD provides a good example because serotonergic alterations are directly related to the severity of disease.

#### 5.1.1. Major Depressive Disorder (MDD)

MDD is defined as a pervasive and persistent low mood with a multi-factor cause. Symptoms have degrees of severity that are associated with changes in both CNS and peripheral 5-HT concentrations [210]. In addition, MDD patients commonly have altered cortisol and cytokine blood levels [210–212]. Lymphocytes from MDD patients express lower levels of SERT in comparison with those from healthy volunteers without changes in the intracellular concentration of 5-HT [52, 64]. There are no changes in SERT expression in monocytes, but the intracellular concentration of 5-HT in monocytes is higher in MDD patients [64]. MDD patient lymphocytes display a three-fold increase in LPS-stimulated proliferation, an effect blocked by 5-HT_1_ antagonists [213]. In addition, there are more 5-HT_2A_ clusters on the lymphocytes of MDD patients, whom are responsive to SSRI treatment [44].

When MDD patients are treated with SSRIs there are changes in lymphocyte subpopulations and in systemic inflammatory mediators (Table 4). Before treatment, MDD patients have higher blood cortisol, IL-4, IL-13, and IL-10 than healthy volunteers [210–212]. After 20 weeks of treatment, concomitantly with a remission of the depressive episode there are increases in IL-2 and IL-1*β* but no change in cortisol levels. At week 52 of treatment there is a significant reduction in cortisol levels with an increase in IL-1*β* and IFN-*γ* and a decrease in anti-inflammatory cytokines [211]. Regarding lymphocyte subpopulations, before SSRI treatment MDD patients had more NK cells compared to healthy volunteers (312 ± 29 versus 158 ± 30; cells/mL), but no differences were found in the T and B cell populations. After 20 weeks of treatment, patients experienced a remission of depressive episodes along with an increase in NK cell and B cell populations, which remained heightened until the 52nd week of treatment [214]. These findings in conjunction with the fact that lymphocytes from MDD patients respond differently than healthy subjects suggest that the general inflammatory response and specific immune subsets are sensitive to systemic levels of 5-HT and changes in those levels induced by SSRIs. However, further studies are still required to fully understand how components of the serotonergic system are differentially expressed on immune cell subsets. This may clarify the mechanisms involved in MDD progression and highlight new therapeutic targets for its treatment.

#### 5.1.2. Fibromyalgia

Fibromyalgia (FM) is a common chronic pain syndrome that primarily affects the joints and muscles and is generally associated with other somatic and psychological symptoms, including fatigue, poor sleep, cognitive difficulties, and stress [214]. FM patients have central sensitization and increasing glial cell activation, which, in turn, favors pain signaling and activates the release of pro-inflammatory cytokines, nitric oxide, prostaglandins, and ROS that sustain the hyperexcitable state of the spinal cord [215–217].

Several neurotransmitters are involved in FM-associated central sensitization. 5-HT_2_ and 5-HT_3_ are involved in pain control, indicating a key participation of the serotonergic system [218, 219]. Levels of 5-HT are low in the serum and cerebrospinal fluid of FM patients and correlate with clinical symptoms [66, 220–222]. FM patients also have increased B cell and decreased NK cell counts [223]. The administration of the 5-HT_3_ antagonist tropisetron [224] or high doses (45 mg) of the SSRI fluoxetine [225] produce analgesic and/or other beneficial effects in FM patients (Table 4), suggesting that regulation of the serotonergic system can be useful. However, to date it is not known whether the components of the serotonergic system can be altered in the immune cells of patients.

#### 5.1.3. Infections

Immune responses to viruses, bacteria, fungi, and parasites all require 5-HT. Human immunodeficiency virus (HIV) infection is a primary model for the study of 5-HT during infection (Table 4). 5-HT controls HIV replication in T4 lymphocytic cell lines [226] and modulates NK cell activation in HIV-infected patients [76]. The virus infects macrophages, which provide a reservoir of infection [227]. 5-HT decreases the expression of the HIV coreceptor CCR5 on infected macrophages and reduces proviral synthesis 50% [228, 229]. These effects can also be achieved with an agonist targeting 5-HT_1_ but not with one of 5-HT_2_ [229]. Furthermore, SHIV-infected PBMCs from Rhesus monkeys (*Macaca mulatta*) have 10 times less SERT mRNA than uninfected controls [230]. The authors of this study suggest that low SERT expression may be responsible for the symptoms of depression found in HIV-patients. SSRI drugs are cytotoxic to NK cells taken from HIV-infected patients [76]. Similarly, SSRIs stimulate macrophage activity* in vivo* and reduce HIV replication in macrophages and T cells [57]. Interestingly, these effects were independent of patients' psychological status indicating that mood changes are not necessary for 5-HT to have an immunomodulatory effect. These findings suggest that components of the serotonergic system may be suitable therapeutic targets for the control of HIV infection.

Patients infected with hepatitis C virus (HCV) and treated with IFN-*γ* have reduced levels of tryptophan and kynurenine [231], suggesting that 5-HT synthesis and systemic concentrations may be reduced. Furthermore, HCV-infected patients given SSRI therapy have lower viral replication rates [232]. Therefore, SSRIs and/or 5-HTR-targeting drugs may be beneficial for many viral infections.

There is also evidence that SSRIs have antibacterial (especially against gram-negative bacteria) [233, 234], antifungal [235], and antiparasitic [79, 236] effects. The available reports establish a direct cytotoxic effect of SSRIs on the pathogen (Box 2). However, it will be interesting to characterize whether immune cells contribute to infection control during SSRI treatment.

**Box 2 (selective serotonin reuptake inhibitors (SSRIs) have antiparasitic and antifungal activity).**

Sertraline and fluoxetina decrease* in vitro* cell viability of* Aspergillus spp.* and* Candida parapsilosis* [235, 237, 238]. Sertraline is likely effective at controlling* Leishmania donovani *infection in a mouse model by inhibition of parasite respiration [79]. Mianserine decreases the motility of* Schistosoma mansoni*, the most common species of schitosomes, and 5-HT receptors are expressed in these helminthes at the larvae and adult stages but are overexpressed once they enter NCS [236]. Together these results demonstrate that parasites and fungi express SERT-like proteins indicating that they are likely sensitive to SSRIs and systemic changes of 5-HT in the host. Furthermore, the serotonergic system in parasites and fungi may constitute a pharmacological target for drug design. A BLAST search using the human sequence of SERT (gen SLC6A4) against the* Aspergillus* taxa (taxid: 5052) in the GeneBank database identified seven conserved hypothetical proteins assigned either as uncharacterized eukaryotic solute carrier 6 (EAU35443.1; XP_682235.1; CBF84552.1; XP_001215815.1; EIT73756.1) or sodium/chloride dependent neurotransmitter transporter (XP_001826855.1; XP_002385196.1).

#### 5.1.4. Alzheimer's Disease

Alzheimer's disease is a neurodegenerative disorder and the primary cause of dementia in elderly people [239]. *β*-amyloid deposits in senile plaques and neuro-fibrillary tangles that affect brain cell function are characteristic of the disease [240]. Symptoms of dementia and depression, which are related to reduced levels of 5-HT, are present in 50–90% of patients [241]. NK cells isolated from patients with Alzheimer's disease have a high density of 5-HT_2C_ compared with cells from late onset depression patients. However, there is no difference in the level of 5-HT_1A_, 5-HT_2A_, and 5-HT_2B_ receptors on PBMCs [72]. The abundant increase of 5-HT_2C_ on NK cells may be a compensatory mechanism for reduced 5-HT availability. Activation of 5-HT_2C_ inhibits NK cell activity, which may partially explain why Alzheimer's disease patients are more susceptible to viral infections [72, 242].

SSRIs have been used to treat depression in Alzheimer's disease patients. In these patients, SSRIs stimulate cell survival mechanisms, cell adhesion, and lymphocyte activation [243]. Similarly, in a murine model of the disease (hAPP/PS1), chronic oral administration of a 5-HT_4_-selective agonist (SSP-002392) reduced *β*-amyloid production and deposition and improved mouse memory [244]. Given the complexity of the cell population expressing 5-HT_4_ in the brain, it is difficult to speculate about a mechanism of action. However, microglial cells may be involved because they express 5-HT_4_ and can phagocytose *β*-amyloid deposits, an activity promoted by agonist [244]. This indicates that 5-HT_4_ agonists induce immunomodulation in microglial cells. It remains to be determined whether similarly activated immune cells influence Alzheimer's disease progression and symptoms.

### 5.2. Diseases with High Systemic Levels of 5-HT

From an immunological point of view, the diseases in this group, such as asthma, arthritis, and cancer, are the result of dysregulated inflammatory responses. Therefore, the association of these diseases with high circulating levels of 5-HT reinforces its role as an immunomodulator.

#### 5.2.1. Asthma

Asthma is a chronic inflammatory disease of the lungs with consequent narrowing of the airways. 5-HT levels are increased in asthma patients and SSRI treatment improves clinical symptoms.* In vitro*, addition of 5-HT or 5-HT_1_/5-HT_2_ agonists to alveolar macrophages increases the production of IL-10, nitric oxide, and PGE-2, but reduces TNF-*α* and IL-12 production. Interestingly, receptor antagonists do not affect secreted-cytokine profiles [177]. Regardless, these results indicate that cytokine production is under the control of 5-HT, and therefore, regulating its systemic concentrations may be useful for asthma patients.

#### 5.2.2. Rheumatoid Arthritis (RA)

Rheumatoid arthritis is a chronic disease that causes pain, stiffness, and swelling that limits the motion and function of many joints. While RA can affect any joint, smaller joints of the hands and feet are most commonly involved. Inflammation can affect organs, such as eyes and lungs, in addition to joints. The 5-HT concentrations in platelet-free blood are 1.6- to 2.3-fold higher in RA patients than in healthy controls (reported average serum concentrations were 1130 nmol/L versus 704 nmol/L, resp.) [245]. This has led to proposals that 5-HT is involved in the pathology, onset, and/or progression of the disease. Treating patients with 5-HT_3_ antagonists combined with intra-articular glucocorticoids has analgesic effects [246]. While there have yet to be any reports on the responses of RA patient immune cells to 5-HT, the role of serotonergic system in arthritis or osteoarthritis has been studied* in vitro* and* in vivo*. An osteoarthritis model using cultured synovial tissue demonstrated that 5-HT stimulation increases the expression of 5-HT_2A_ and 5-HT_3_ as well as the release of PGE-2 into the medium. The addition of receptor antagonists inhibits PGE-2 production [74], which may explain the beneficial effects seen in arthritis patients given this treatment [74].

SSRIs can also affect arthritis development. Fluoxetine and citalopram inhibit disease progression in a collagen-induced mouse model of arthritis as well as in human RA synovial membranes cultures. In addition, macrophages from RA patients display impaired TLR-3, -7, -8, and -9 signaling after SSRI exposure [247]. Therefore the evaluation of SSRIs in RA patients is of interest.

#### 5.2.3. Cancer

While the role of serotonergic system in cancer patients has not been largely studied, advanced stages of breast cancer correlate with increased levels of systemic 5-HT [248]. In mouse models of melanoma and lymphoma, SSRI treatment reduced tumor growth by 50%, inhibited IL-10 and IFN-*γ* production, and increased IL-1*β* production [249]. Similarly, exposing a Burkitt's lymphoma cell line to different SSRIs (fluoxetine, paroxetine, or citalopram) decreased DNA synthesis and induced cell death [250]. Although further studies are required, these results suggest that the serotonergic system can impact cancer cells directly or indirectly through immune cell activation.

## 6. Conclusions

Given the importance of 5-HT as a neurotransmitter, studies of the serotonergic system have primarily been limited to the CNS. Recently, however, a large amount of experimental evidence indicates that the serotonergic system has important physiological roles in the immune, vascular, and digestive systems. In this review we discussed the immunomodulatory effects that 5-HT can induce by activating 5HTR and SERT, which are differentially expressed on many leukocytes. These effects can be variable depending on cellular phenotype. For example, 5-HT induces dose-dependent cytoskeletal reorganization and diapedesis during chemotaxis as well as granule secretion in granulocytes and myeloid cells. In comparison to these non-transcriptional responses, 5-HT regulates cell proliferation and cytokine production at the transcriptional level in leukocytes.

The human serotonergic system is complex and comprised of many elements. It includes 18 genes, including 5HTRs and one SERT, several of which have multiple isoforms (creating at least 10 additional proteins). Furthermore, the receptor signaling-transduction system that regulates 5-HT responses involves a large number of genes [251, 252] providing several points of regulation depending on cellular phenotype. In conclusion, cells of the immune system express transduction machinery that does not necessarily overlap with that in the CNS. This allows for differential responses to the same 5-HT ligand within the immune and nervous systems.

The information presented here is based on existing reports, but we must consider that many early studies of 5-HT receptors used primarily pharmacologic approaches and the results are sometimes not supported by more recent genetic approaches. For example, although early studies suggest role of SERT in T cells, genetic studies suggest that T cells express DAT (dopamine but also low affinity 5-HT transporter) [253, 254]. Although the effects of 5-HT on the immune system requires further characterization, it is logical to anticipate altered immune responses in patients with dysregulated serotonergic systems. For these patients, experimental evidence suggests that SSRI or 5HTR antagonist treatment may provide beneficial immunomodulatory effects.

## Figures and Tables

**Table 1:** Serotonergic proteins expressed in human immune cells.

	5HT_1_	5HT_2_	5HT_3_	5HT_4_	5HT_5_	5HT_6_	5HT_7_	SERT	Anabolism	Catabolism
Cellular Types	1A: B Lymphocytes^∗‡^ [35, 36] 1A: T Cell^§∗†^ [37] 1A: Mast cells^§∗‡^ [38] 1A: Eosinophils^§‡^ [39] 1B: Eosinophils^§‡^ [39] 1B: Dendritic cells^§‡^ [2] 1B: Mast cells^§∗‡^ [38] 1B: T cell^∗†^ [40, 41] 1D: Mast cells^∗‡^ [38] 1E: Monocytes^§‡^ [42] 1E: Eosinophils^§‡^ [39] 1E: Dendritic cells^§‡^ [2, 43] 1E: Mast cells^§‡^ [38]	2A: Lymphocytes^§†^ [44] 2A: Monocytes^§‡^ [42] 2A: PBMC^§†^ [10] 2A: Dendritic cells^§‡^ [2] 2A: Mast cells^§∗‡^ [38] 2A: Eosinophils^§∗‡†^ [39, 45] 2A: T cell^∗‡†^ [40] 2B: Mast cells^§∗‡^ [38] 2B: Dendritic cells^§‡^ [2, 43] 2C: Mast cells^§‡^ [38]	3: Mast cells^§‡^ [38] 3: Dendritics cells^§‡^ [2, 43] 3A: Lymphocytes^§‡†^ [46] 3A: Monocytes^§‡†^ [42, 47] 3A: B cells^§‡†^ [48] 3A/3E: Lymphatic Ganglion^§‡^ [49]	Dendritic cells^§‡^ [2, 43] Monocytes^§‡^ [42, 50] Mast cells^§‡^ [38]		Eosinophils^§‡^ [39] Mast cells^∗‡^ [38]	Dendritic cells^§‡†^ [2, 43, 50] Monocytes^§‡^ [42, 50] Mast cells^§∗‡^ [38] T Cell^†‡∗^ [40]	Lymphocytes^§∗‡†^ [51–55] Leukocytes^§‡^ [56] Monocytes^§†^ [57] Dendritic Cell^§∗‡†^ [58, 59]	TPH1: T Cells^‡∗^ [40, 58] TPH1: Mast Cell^∗‡^ [38]	IDO: Lymphocytes^§‡†^ [60] IDO: Monocytes^§‡†^ [61] IDO: Dendritic Cells^§‡†^[60] MAO-A: Monocytes^§‡^ [62]

^§^Human, ^∗^Murine, ^‡^Expression, ^†^Protein.

**Table 2:** The effect of 5-HT on MDDC cytokine production.

Cytokine secretion	5-HT receptors involved	References
↑ IL-6	5HT_3_, 5HT_4_ and 5HT_7_	Müller et al., 2009 [43]
↓ IL-12p70	5HT_4_ and 5HT_7_	Müller et al., 2009 [43]
↑ IL-10	5HT_4_ and 5HT_7_	Müller et al., 2009 [43]
↑ IL-8, IL-1*β* ↓ IL-12, TNF-*α*	5HT_3_, 5HT_4_ (G*α*s-coupled), and 5HTR7 (G*α*s-coupled)	Idzko et al., 2004 [2]

**Table 3:** Pathology-associated serotonergic protein expression in immune cells.

Patology/condition	5-HT, SERT and 5HTR variation	Reference
Major depression disorder Human	↓ SERT in platelets ↓ 5-HT serum levels ↓ SERT in lymphocytes	Mössner et al., 2007 [63] Fazzino et al., 2008 [64] Lima and Urbina, 2002 [52] Peña et al., 2005 [65]

Fibromyalgia Human	↓ 5-HT serum levels ↓ SERT platelets	Bazzichi et al., 2006 [66]

Schizophrenia Human	↑ 5HT_2A_ (polymorphism T102C) in lymphocytes ↑ 5HT_3A_ (gene) in PMBC ↓ SERT (re-uptake) in lymphocytes	Williams et al., 1997 [67] Abdolmaleky et al., 2004 [68] Shariati et al., 2009 [69] Marazziti et al., 2006 [70]

Asthma Human	↑ 5-HT plasma levels	Lechin et al., 1998 [71]

Alzheimer disease Human	↑ 5HT_2C_ in NK cells ↓ 5-HT serum levels	Martins et al., 2012 [72]

Psoriasis Human	↑ SERT of skin biopsies, dendritic cells	Thorslund et al., 2013 [59]

Alcohol exposition 0.1% 24 h Culture, dendritic cells	↑ SERT in dendritic cells ↓ 5-HT extracellular levels	Babu et al., 2009 [73]

Arthritis Cell culture	↑ 5HT_2A_ mRNA in macrophage-like synovial cells ↑ 5HT_3_ mRNA in macrophage-like synovial cells	Seidel et al., 2008 [74]

Mitogen activation with concanavalin Rat lymphocyte	↑ 5HT_7_ mRNA	Urbina et al., 2014 [75]

**Table 4:** Serotonergic drugs and their effects on the immune system.

Pathology/treatment	Result	Reference
HIV chronic infection/ SSRI (citolopram) Human	↓ Macrophage infectivity ↓ Viral replication in macrophages and T cells ↑ NK cell activity	Benton et al., 2010 [57] Evans et al., 2008 [76]

HCV infection IFN-*α* and SSRI (escitalopram) Human	↓ Depression symptoms	Schaefer et al., 2012 [77]

Tendinopathy and facial pain 5-HT3 antagonist (tropisetron) Human	Analgesic effect	Müller and Stratz, 2004 [78]

Infection with *Leishmania donovani* SSRI (sertraline) Balb/c mice	SSRI killed *L. donovani* promastigotes and intracellular amastigotes	Palit and Ali, 2008 [79]

CT26/luc colon carcinoma-bearing mice Mirtazapine	↓ T cell infiltration in tumor	Fang et al., 2012 [80]
